# *Yersinia pestis* Orientalis in Remains of Ancient Plague Patients

**DOI:** 10.3201/eid1302.060197

**Published:** 2007-02

**Authors:** Michel Drancourt, Michel Signoli, La Vu Dang, Bruno Bizot, Véronique Roux, Stéfan Tzortzis, Didier Raoult

**Affiliations:** * Université de la Méditerranée, Marseille, France; † Ministère de la Culture, Paris, France; ‡ Service archéologique, Martigues, France

**Keywords:** *Yersinia pestis*, plague, paleomicrobiology, dental pulp, dispatch

## Abstract

*Yersinia pestis* DNA was recently detected in human remains from 2 ancient plague pandemics in France and Germany. We have now sequenced *Y. pestis glp*D gene in such remains, showing a 93-bp deletion specific for biotype Orientalis. These data show that only Orientalis type caused the 3 plague pandemics.

Three historical pandemics have been attributed to plague. The causative agent, *Yersinia pestis*, was discovered at the beginning of the ongoing third pandemic. The etiology of the 5th-7th–century first pandemic and the 14th-18th–century second pandemic, however, remained putative until recently (*1*). Indeed, results of 16S rRNA gene-based detection using teeth collected from 64 persons’ remains in 7 northern Europe sites remained negative (*2*). When using different molecular targets and the dental pulp as a suitable specimen, we detected *Y. pestis*–specific DNA fragments in European skeletons of persons suspected of having historical plague (*3*–*5*). Our results were independently confirmed on 6th-century Bavarian teeth (*6*). *Y. pestis* comprises biotypes Antiqua, Medievalis, and Orientalis, recognized on the basis of the conversion of nitrate to nitrite and fermentation of glycerol. A fourth biotype, Microtus, describes Medievalis isolates lacking arabinose fermentation. In 1951, Devignat proposed that each of the first 3 biotypes determined each plague pandemic (*7*). This hypothesis was challenged by our multispacer-typing detection of an Orientalis-like biotype in 5th- to 14th-century dental pulp specimens (*5*). A 93-bp deletion from the *Y. pestis glp*D gene encoding the glycerol-3-phosphate dehydrogenase determines lack of glycerol fermentation of the Orientalis biotype (*8**,**9*). Isolates of the other biotypes lack this deletion (*8*). Here, we establish role of Orientalis biotype in the 3 pandemics by sequencing the *glp*D gene from additional ancient dental pulp specimens.

## The Study

We had historical evidence that 3 mass graves excavated in France were used to bury bubonic plague victims. In Vienne, 12 skeletons, including 5 children, buried within the ruins of a Roman temple have been dated from the 7th–9th centuries both by a 5th-century coin and ^14^C dating. In Martigues, 205 skeletons buried in 5 trenches were dated from 1720 to 1721 on the basis of coins and detailed parish bills that listed the victims (Figure). In Marseille, 216 skeletons buried in a huge pit dated from a May 1722 epidemic relapse. We previously confirmed the diagnosis of plague at this site (*3*). Eighteen teeth from 5 skeletons in Vienne, 13 teeth from 5 skeletons in Martigues, and 5 teeth from 3 skeletons in Marseille were processed for the search for *Y. pestis* DNA in the dental pulp. The teeth were processed according to published criteria for authenticating molecular data in paleomicrobiology (*10*): 1) there should be no positive control; 2) negative controls, as similar as possible to the ancient specimens, should test negative; 3) a new primer sequence targeting a genome region not previously amplified in the laboratory should be used (suicide PCR); 4) any amplicon should be sequenced; 5) a second amplified and sequenced target should confirm any positive result; and 6) an original sequence that differs from modern homologs should be obtained to exclude contamination.

**Figure:**
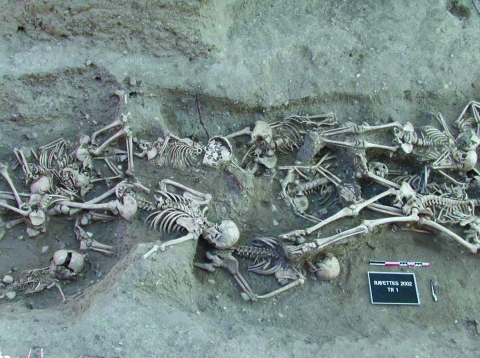
Skeletons from a mass grave in Martigues, 1720–1721, yielded molecular evidence for the *Yersinia pestis* Orientalis biotype. Photograph: S. Tzortzis

Accordingly, DNA samples were submitted for suicide-nested PCR conducted by using 1 negative control (18th-century teeth from skeletons of persons without anthropologic and macroscopic evidence of infection) for every 3 specimens. Two microliters (1 μL for nested PCR) DNA were amplified in a 50-μL mixture containing 10 pmol of each primer, 200 μmol/L each deoxyribonucleotide triphosphate (Invitrogen, Cergy-Pontoise, France), 1.5 U *Taq* polymerase (Invitrogen), and 2.5 μL of a 50-mmol/L solution of MgCl_2_ in 1× *Taq* buffer. Nested PCR aimed to encompass the entire *glp*D gene incorporated primers: glpD-F1: 5′-GGC TAG CCG CCT CAA CAA AAA CAT-3′ (positions 170080–170103, reference: *Y. pestis* strain CO92 genome sequence AJ414159.1)/glpD-R2: 5′-GGT GCC AGT TTC AGT AAC AC-3′ (positions 170402–170383) for initial PCR and glpD-F3: 5′-CGC TGT TTC GAA CAT TCA GA-3′ (positions 170230–170249) /glpD-R3: 5′-GGC CAA GGC TTC ACT TAC CA-3′ (positions 170373–170354) for nested PCR. PCRs were performed in a T3 thermocycler (Biolabo, Archamps, France) under the following conditions: an initial 2 min of denaturation at 95°C was followed by 43 cycles (38 cycles for nested PCR) of denaturation for 30 s at 94°C, annealing for 30 s at 58°C, and extension for 90 s at 68°C. The amplification was completed by holding the reaction mixture for 7 min at 68°C. PCR products purified by using a MultiScreen PCR plate (Millipore Corp., Bedford, MA, USA) were sequenced with a DNA sequencing kit (Big Dye Terminator Cycle Sequencing V2.0; PE Biosystem, Courtaboeuf, France) and subjected to electrophoresis with the 3100 Genetic Analyzer (Applied Biosystems, Foster City, CA, USA). The sequences were compared in the GenBank database (www.ncbi.nlm.nih.gov/GenBank) using the multisequence alignment Clustal within the BISANCE environment.

No amplification was observed in 11 negative controls, but 5 of 36 teeth yielded an amplicon of 191-bp length in 2 of 4 persons’ remains from Vienne, 2 of 5 from Martigues, and 1 of 3 from Marseille. Amplicons exhibited 100% sequence similarity with that of the *Y. pestis* Orientalis *glp*D gene (GenBank accession nos. AY312359 for tooth 35–0235, Vienne; DQ073797 for tooth SQ401521 and DQ073798 for tooth SQ408113, Martigues; and AY312360 for tooth 25–0225, Marseille) and were characterized by a 93-bp deletion when compared with the *glp*D gene sequence of the *Y. pestis* Medievalis biotype (GenBank accession no. AE 013994).

## Conclusions

In this study, contamination of the ancient specimens is unlikely because of the extensive precautions we took, including use of the suicide PCR protocol excluding positive controls (*4*). Accordingly, *glp*D gene had never been investigated in our laboratory before this study, and negative controls remained negative. The specificity of the amplicons was ensured by complete similarity of experimental sequences with that of the *Y. pestis* Orientalis *glp*D gene (*8*). One site (Marseille, 1722) was previously positive for *Y. pestis* after sequencing of 2 different targets (chromosome-borne *rpob* and plasmid-borne *pla* genes) in other specimens collected in other persons’ remains (*3*).

These results therefore confirm the detection of *Y. pestis*–specific DNA in plague patients’ remains from the first and second epidemics (*3*–*6*). We observed a 93-bp in-frame deletion within the *glp*D gene sequences obtained from ancient dental pulp specimens. This deletion has been found only in Orientalis biotype isolates in 2 independent studies comprising a total of 77 and 260 *Y. pestis* isolates, respectively, of the 4 biotypes (*8**,**9*).

After previous demonstration of *Y. pestis* Orientalis-type multiple spacer type sequences in Justinian and medieval specimens (*5*), we now have cumulative evidence using 2 different molecular approaches that *Y. pestis* closely related to the Orientalis biotype was responsible for the 3 historical plague pandemics.
